# Bond strength and adhesive interfacial micromorphology of self-adhesive resin cements: Effect of reduced times of pre-etching

**DOI:** 10.4317/jced.56120

**Published:** 2019-11-01

**Authors:** Fereshteh Shafiei, Sahba Kamran, Mahtab Memarpour, Tayebeh Aghaei

**Affiliations:** 1 Oral and Dental Disease Research Center, Department of Operative Dentistry, School of Dentistry, Shiraz University of Medical Sciences, Shiraz, Iran; 2 Student Research Committee, School of Dentistry, Shiraz University of Medical Sciences, Shiraz, Iran; 3 Oral and Dental Disease Research Center, Department of Pediatric Dentistry, School of Dentistry, Shiraz University of Medical Sciences, Shiraz, Iran

## Abstract

**Background:**

Although acid-etching could increase enamel bond strength of self-adhesive (SA) cements, it may lead to negative effect on dentin. The aim of this study was to assess the effect of shortening of phosphoric acid pre-etching duration on enamel/ dentin shear bond strength (SBS) and interfacial microstructure of Panavia SA Luting Plus (PASA) and Bifix SE (BXSE).

**Material and Methods:**

For both enamel and dentin, effect of pre-etching time was significant (*p*<0.001); however, effect of cement brand and the interaction of two factors was significant only for dentin (p≤0.008). Three pre-etching times increased SBS of both cements to enamel and BXSE to dentin. In case of dentin SBS of PASA, this improvement was significant only for 5 and 10 seconds (*p*<0.001). Adding acid-etching in the three times to both cements similarly improved interfacial adaptation, particularly for enamel.

**Results:**

The evaluations of the biofilm elimination showed results that indicate that the 4% sodium hypochlorite group with positive pressure irrigation presented significant differences with the group that had irrigation with sodium hypochlorite activated with XP-endo Finisher and the chlorhexidine groups to 2% (*P*<0.05).

**Conclusions:**

Interfacial bond strength and adaptation of SA cements used in this study were improved following addition of enamel/dentin etching step for a short time. This was not exceeded for longer times than 5-second.

** Key words:**Dentin bond strength, enamel bond strength, pre-etching time, self-adhesive cement.

## Introduction

Cementation of indirect restorations is critical step to achieve durable successful treatment (1,2). Technique sensitivity and complex procedures involved in adhesive system/conventional resin cementation lead to introduce simplified self-adhesive (SA) resin cements contained acidic modified monomers (3,4). In addition to easy applying property, clinicians could benefit from low moisture-sensitivity, reduced post-operative sensitivity (1,5) and incompatibility of dual-cure cement with acidic adhesives (6). However, this simplifying may compromise bonding effectiveness due to their limited etching potential and superficial interaction of the high-viscous cement with tooth structure, especially enamel (7-10).

For many years, phosphoric acid-etching of enamel establish stable, high enamel bonding (11,12). A number of studies have been documented reliable enamel bond strength of SA cements after acid-etching (1,3,6,7,12,13). However, effect of acid-etching on dentin bond strength is controversial (6,14), with some reports demonstrating its adverse effect on dentin (1,3,7). In the reported studies, although acid-etching time was 15 seconds for dentin(4), this time varied from 15 to 30 and 60 seconds for enamel (3,12,13), in clinical practice, enamel etching for these durations without etching of prepared dentin in a cavity is difficult to control, leading to over-etching adjacent dentin. Use of shorter etching time might help to achieve adequate enamel bond without overexposure of collagen matrix that could not be penetrated by high-viscous SA resin cement. Subsequently, the non-infiltrated collagen might result in creation of the adhesive interface being more vulnerable to biodegradation (7).

Recently, shortening acid-etching time from 15 seconds to 5 or even 3 seconds has been found to provide higher enamel bond strength of one-step self-etching adhesives compared with non-etching method (15). Furthermore, unlike to 15-second etching, this reduced etching time was capable of improving dentin bond strength of universal adhesives contained functional monomers (16). Nevertheless, when compared to adhesive systems, SA cements have a high viscosity with lower wetting ability. This might cause their different behavior to shortened pre-etching time. So far, no study has been evaluated weather reduced acid-etching time enable to provide high enamel bond strength of SA cements while minimizing the negative effect on dentin, even enhancing bonding ability. Therefore, this study was aimed to test the null hypothesis that there are no significant differences between adhesive interfacial micromorphology and bond strength of two SA cements to enamel and dentin that acid-etched for different duration times, 15, 10, 5 and 0 seconds. 

## Material and Methods

Sound extracted human third molars were collected under informed consent and used in this experiment following the protocol approval by Shiraz University of Medical Sciences Research Research Ethics Committee. The selected teeth were stored in disinfectant solution at 4◦C and then distilled water until preparation for cement bonding. The roots were removed from the teeth and discarded. After mesiodistally sectioning 48 teeth and embedding in acrylic resin molds, their buccal and lingual surfaces were grounded using wet 320-grit and 600-grit abrasive papers to expose a flat enamel area covered with a standardized smear layer, providing 96 enamel specimens. The same steps were performed to provide a flat smear layer-covered dentin area on occlusal surface of 96 teeth. The surfaces were examined under a stereomicroscope to verify the lack of any remaining enamel.

The prepared enamel and dentin surfaces were rinsed and kept moist until and during the experiment. Each of them were randomly divided into two SA cement groups used, then were subdivided into four groups according to prior acid-etching used as follows: 1) non-etch group, 2) acid-etching for 5 seconds, 3) acid-etching for 10 seconds, 4) acid-etching for 15 seconds. Acid-etching was 35% phosphoric acid (Vococid, VOCO, Cuxhaven, Germany) and two SA cements were Panavia SA Luting Plus (PASA, Kuraray, Okayama, Japan, Lot≠ 170010) and Bifix SE (BXSE, Voco, Lot≠ 1714143). The two pastes of each cement were mixed according to manufacturers’ instruction and inserted on the surface confined by a cylindrical plastic mold with internal diameter of 3 mm and height of 2 mm (17). The cement was under a standardized pressure (454 gr) to simulate seating pressure. Thereafter, the cement was light-cured from each side for 40 seconds using a light-curing unit (VIP Junior, Bisco, Schaumburg, IL, USA) operating at 600 mW/cm² light intensity. The cemented specimens were stored in distilled water at 37° C for 7 days to complete cement setting.

The specimens were then submitted to shear loading through a knife-shaped indentor in a universal testing machine (Zwick Roell, Z020, Ulm, Germany) at a crosshead speed of 1mm/min. The shear bond strength (SBS) was recorded in Megapascal (MPa). Data from each substrate (enamel/dentin) were statistically analyzed using a separate two-way ANOVA and then by Tukey test (α=0.05).

The debonded surfaces were assessed using a stereomicroscope to classify the failure mode as follows: A) adhesive failure at the tooth-cement interface, C) cohesive failure in tooth structure or cement, M) mixed failure, mixed of both adhesive and cohesive failures.

Field-emission scanning electron microscopy (Fe-SEM) preparation: An additional specimen from each enamel/dentin group of each cement was prepared to observe their interfacial morphology under field emission-scanning electron microscopy (Fe-SEM). Following embedding in epoxy resin, the cemented specimen was sectioned to expose the interface. The sectional surfaces were wet-polished with a series of abrasive discs and finally with a diamond paste. They were demineralized with phosphoric acid and then cleaned, followed immersed in a sodium hypochlorite solution. After rinsing, a dehydration process was performed on them. The interfacial surfaces were then coated with gold in a vacuum evaporator and observed with Fe-SEM (MIRA 3, TESCAN, Brno, Czech Republic).

## Results

The results of the enamel and dentin SBS of the two SA cements affected by non-etching and the three different etching times are presented in  Table 1.

**Table 1:**
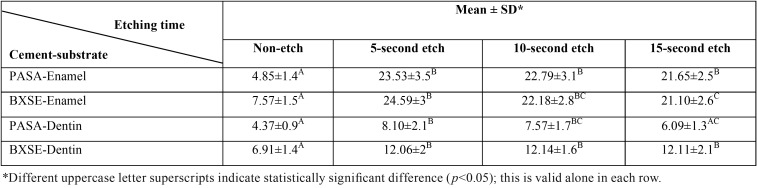
Mean shear bond strength and standard deviation (in MPa) of two cements to enamel and dentin in the four etching groups.

According to two-way ANOVA, SBS to enamel was significantly affected by etching time (*p*<0.001); the effect of cement brand and the interaction of the two factors were not significant (*p*<0.2). Tukey test revealed that all the etching times significantly increased SBS of PASA similarly. However, in case of BXSE this improvement was not the same for these times; 5-second pre-etching revealed a significantly higher SBS than that achieved with 15-second.

SBS to dentin was significantly affected by two variables (etching time and cement) (*p*<0.001) and their interaction was also significant (*p*=0.008). For PASA cement, 5- and 10-second etching significantly increased SBS to dentin (p<0.001), whereas 15-second etching did not (*p*=0.057). For BXSE cement, 5- , 10- and 15-second pre-etching resulted in a higher SBS to dentin compared to non-etch group (*p*<0.001). There was no difference between the three etched groups.

Evaluation of fractured surfaces revealed that for both cements, adhesive failure was the main failure mode in the non-etch group (control) whilst mixed failure was frequently observed in the three etching groups ( Table 2).

**Table 2:**
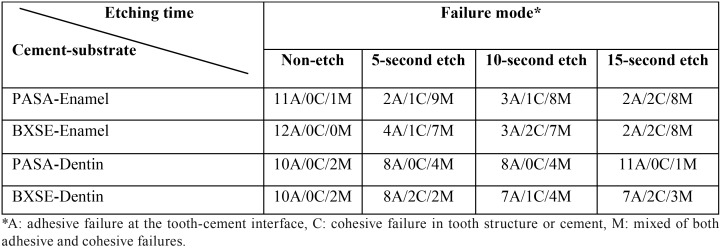
Failure modes distribution of the two cements- enamel and dentin bonded groups in the four etching times.

-Fe-SEM observations

**Figure 1:**
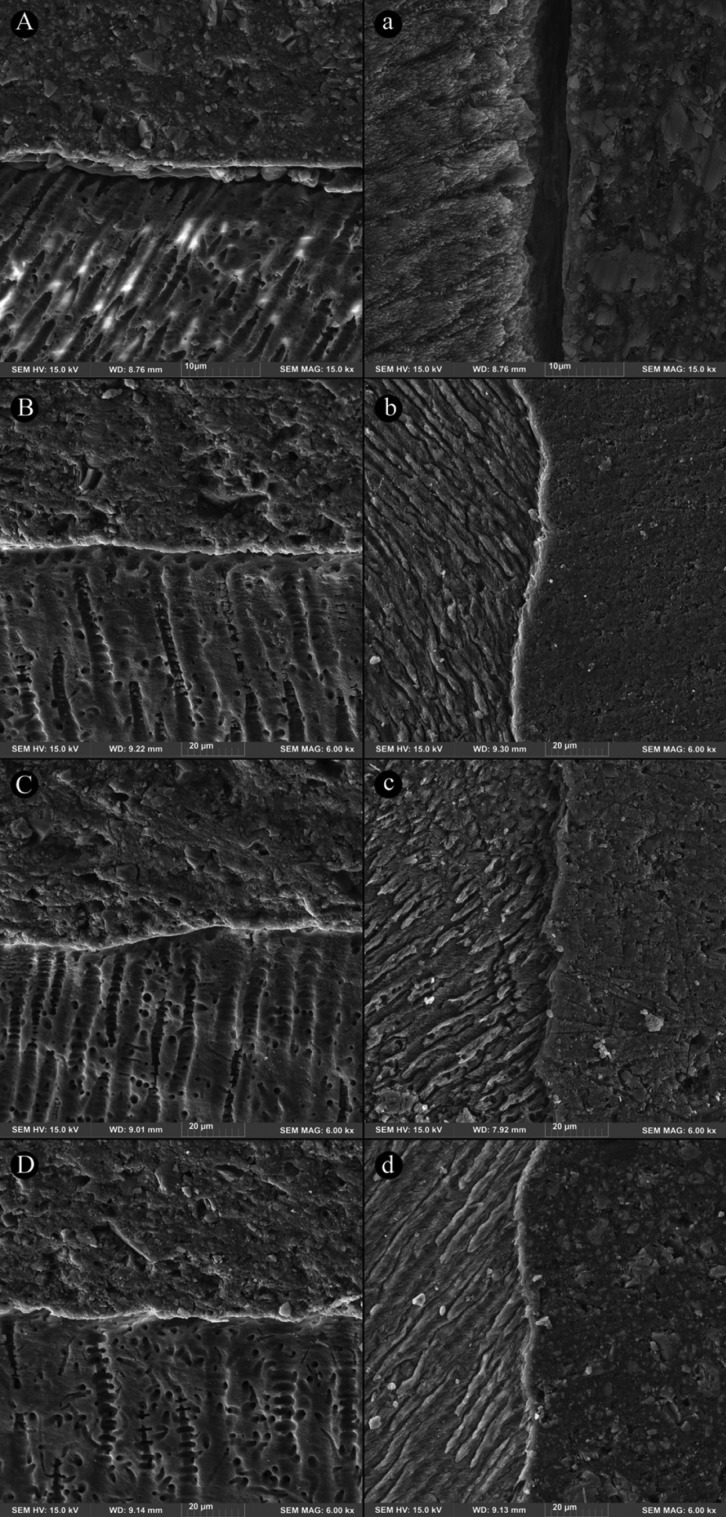
Fe-SEM images of dentin-cement interface (A-D) and enamel-cement interface (a-d) of PASA cement in non-etch, 5-second etching, 10-second etching and 15-second etching, respectively: A) a relative well-adapted interface with no distinct hybrid layer, a) an evident discontinuity at enamel interface; B) an intimate interface with a very thin hybrid layer, b) a continuous interface with several resin penetrations; C) a well-adapted interface, c) a well-adapted interface; D) an intimate interface, d) continuous interface with very short resin extensions.

**Figure 2:**
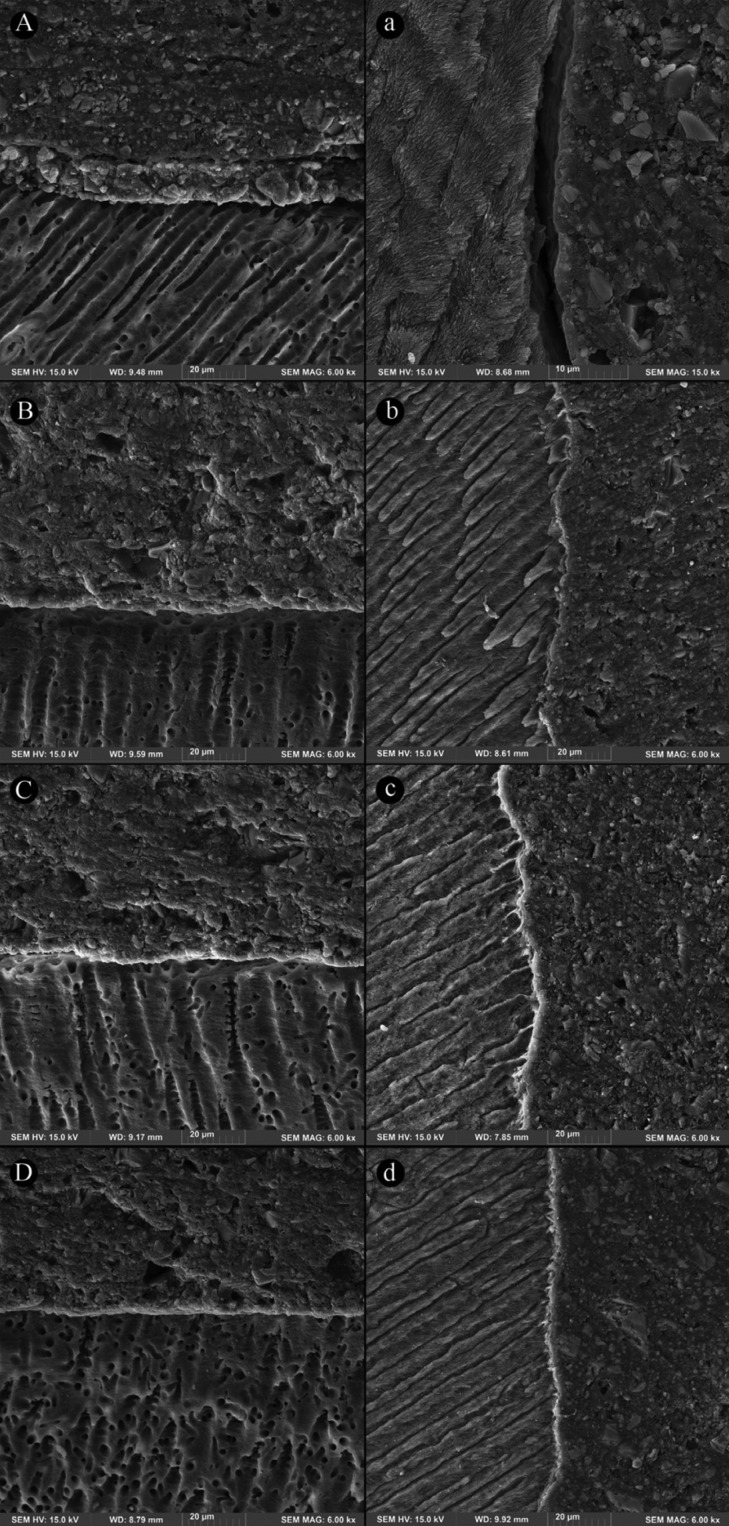
Fe-SEM images of dentin-cement interface (A-D) and enamel-cement interface (a-d) of BXSE cement in non-etch, 5-second etching, 10-second etching and 15-second etching, respectively: A) a relative well-adapted interface, a) an evident large gap at enamel interface; B) an intimate interface with a very thin hybrid layer, b) a continuous hybridized interface with several resin penetrations; C) a well-adapted interface with a few very short resin tags, c) a well-adapted interface; D) an intimate, lightly hybridized interface , d) continuous interface with very short resin extensions.

The dentin/enamel-cement interfacial micromorphology of PASA and BXSE in the four groups (non-etch and three acid-etch) was shown in Figures 1 and 2, respectively. For both cements, the enamel interface of non-etch (control) group exhibited a large discontinuity in a higher magnification that after adding pre-etching step, was not seen in the three etched groups. In these groups, a thin hybrid layer along with signs of resin infiltration was noticeable.

A relatively intimate adaptation with no clear formation of hybrid layer at the both cement-dentin interfaces was illustrated in the non-etch groups. In the three etched groups, this interface was continuous, well-adapted, showing a very thin hybrid layer and a few resin tags with very short length.

## Discussion

Despite high attraction to simplify and shortening luting procedures, creation of high stable bond to tooth structures is mandatory to guarantee successful cemented restorations (3). According to the results of this study, acid-etching enamel improved SBS of both SA cements; shortened etching times were as effective as 15-second etching for PASA, in case of BXSE, even 5-second etching was more effective than that of 15-second etching. Therefore, in case of enamel, the null hypothesis was rejected. Although no study has assessed these reduced etching times, similar beneficial effect of enamel acid-etching for 15 seconds or longer times (30 and 60 seconds) has been reported (3,6,7,12,13). Since hydrophobic smear layer covering surface could hinder its wettability, this improvement was attributed to removal of the smear layer (12,15) and exposure of the hydroxyl groups of the enamel, converting hydrophobic surface to hydrophilic surface (18). This property is compatible with initial intrinsic hydrophilicity of SA cements due to their acidic monomers content and water formed during neutralization processes (1,4,19). Increased enamel wettability/surface free energy and polarization of enamel result in better chemical interaction between the acidic monomers and minerals (12,15). This might help to form micromechanical interlocking through slight infiltration into the tooth structures (19,20). The surface modifications may be provided by the acidic monomers following smear layer removal by acid etching for a short time, ie 5 seconds. Extending the etching from 3 seconds to 15 seconds was indicated not to provide an additional increase in total surface free energy (15).

Although increasing etching time was reported to create rougher surface, this roughness does not necessarily lead to higher bond strength (21). The adequacy of a shorter etching time, 3 seconds has been recently found to establish high enamel bond strength of SE adhesives. This result was verified in similar morphologic images of enamel-adhesive interfaces after 3- , 5- , 10- and 15-second etching (15). It was thought that prolonging the etching time may increase depth of surface porosities whilst not alter surface properties (15,22); the latter and chemical bonding of acidic monomers to minerals play more important role in enamel bonding (22). On the etched enamel, the partial eroded crystals and porous surface was demonstrated to enhance the chemical bonding of functional monomers (15). Moreover, the additional chemical bonding of 10-methacryloyloxydecyl dihydrogen phosphate (MDP) of primer and hydroxyapatite crystals of etched enamel (15 seconds) was found to increase immediate and long-term bond strength (23).

For dentin groups of this study, the bonding performance of the two SA cements differed in response to reduced etching time. PASA benefited from 5- and 10-second etching, not from 15-second, whereas for BXSE, all of the etching times yielded a similar beneficial effect. This varied behavior was related to difference in their chemical composition. PASA contain a functional acidic monomer (MDP), with strong chemical bonding ability (24) that not exist in BXSE. It was assumed that a high mineral content of dentin following a short-time etching would provide a suitable substrate for this chemical interaction, while 15-second etching may not remain adequate minerals following extensive removal of them. Nevertheless, dentin bond strength of BXSE was higher than PASA. Their different mechanical properties could be responsible for this finding. In addition, this long etching time might remain a thick and compact collagen network into which the high viscous cement could not have infiltrated. This was explained by De Munck *et al.* and Hikita *et al.* in their study that 15-second acid-etching negatively affected dentin bond strength of a SA cement (Rely X Unicem) (3,13). However, in consistent with our results, 15-second etching increased bond strength of two SA cements with no effect on the other one (14). And no effect was observed for PASA was similar to a recent study on Rely X U100 and U200 (6). These reported variations could be attributed to different properties of used SA cements. Since these are considered as a heterogeneous unit, these effects could have been product-specific (25). 15-second etching was reported to remove the smear layer, roughen surface, and facilitate penetrating the acidic monomer into the demineralized dentin. The increased water content on acid-etched dentin needed for SA cement ionization may be a contributing factor (14). However, the reduced etching time (5 seconds) was speculated to be enough for the high viscous cement, especially PASA with low spreading ability. However, no study has focused on reducing etching time. In light of the attained results for both enamel and dentin surfaces, in clinical situation with both surfaces involvement, reducing pre-acid etching time to 5 seconds for all cavity walls could be suggested to achieve higher bond strength of both SA cements used in this study. In case of PASA, this time was as effective as 15 seconds for enamel and was more effective than that of 15 seconds for dentin. In case of BXSE, 5-second pre-etching was more effective than 15 seconds for enamel and was as effective as 15 seconds for dentin. When compared to 15-second pre-etching, 5-second etching is still time-saving and associated to limited technique-sensitivity in relation to over-etching dentin and consequent possible negative effect on bonding durability.

The microscopic findings observed in this study could confirm the beneficial effect of pre-etching on the cement-tooth, particularly cement-enamel interface for the three etching times. So that a large gap was evident at the cement-enamel interface of non-etch specimen, whilst a thin hybrid layer along with slight penetrating into etched-enamel surfaces was observed for the three etching time groups. However, this microscopic appearance did not differ between the etched groups. These features were supported by higher SBSs recorded in the etched groups of both cements. At the cement-dentin interface, a relatively good adaptation with no distinct hybrid layer was seen at the interface of both cements without pre-etching; following prior acid-etching for the three times, an intimate continuous adaptation with a very thin hybrid layer and a few very short resin tags was detected. This was associated to the increased SBS of all the etched groups, except of 15-second etching for PASA. These observations for non-etch specimens of both SA cements were in accordance with previous interfacial morphologic studies (8,26,27). In one of them, presence of exposed enamel prisms, no hybridized enamel at the interface of the SA cement was displayed (27). This was attributed to very superficial interaction with highly mineralized enamel and effect of polymerization shrinkage stress of the cement on the adhesive interface (28). The beneficial effect of 15-second etching on dentin interface of the other SA cements as longer resin tags was indicated by Pisani-Proenca *et al.* (14).

In this short-term study no adverse effect of 15-second pre-etching on dentin bond strength of SA cements was observed. However, it can be thought that this long pre-etching time remain non-impregnated collagen layer due to inability of the high viscous cement to reach to full depth of demineralized dentin. Shortening etching time may prevent to create this incomplete interface; hence, preserving bond strength over time. This idea should be assessed in longitudinal studies in future.

Although some intra-oral conditions such as applying seating pressure during setting the cement and moisture maintaining the teeth during experiment were simulated in this study, effects of long-term water storage, thermal and load cycling and pulpal pressure were not included. Moreover, SA cements were bonded to a flat dentin/enamel surfaces that reduce effect of polymerization shrinkage of the cements on the adhesive interface. Therefore, this study was considered a preliminary study on reducing pre-etching time and its results should be confirmed in further long-term *in-vitro* and *in-vivo* researches prior to be suggesting as a reliable approach.

## Conclusions

In light of the attained findings, it can be concluded that phosphoric acid-etching prior to SA cementation led to improved interfacial strength and adaptation. Etching for 5-second was as effective as or more effective than 15-second etching for dentin and enamel surfaces, depending on the SA cements composition. Therefore, pre-etching for a reduced time applied for both substrates in one cavity might be suggested, while this is time-saving and less technique-sensitive approach to some extent compared to longer time of pre-etching.
